# Impact of modifiable lifestyle risk factors for prostate cancer prevention: a review of the literature

**DOI:** 10.3389/fonc.2023.1203791

**Published:** 2023-09-08

**Authors:** Francesco Ziglioli, Annalisa Patera, Gianmarco Isgrò, Davide Campobasso, Giulio Guarino, Umberto Maestroni

**Affiliations:** ^1^ Department of Urology, University Hospital of Parma, Parma, Italy; ^2^ Department of Urology, James Cook University Hospital, Middlesbrough, United Kingdom

**Keywords:** prostate cancer, risk factors, prevention, lifestyle, prevention policy

## Abstract

**Introduction:**

Although prostate cancer (PCa) is one of the most common cancers among men, the impact of modifiable risk and protective factors is still being debated. This review aims to qualitatively summarize the most recent studies related to potential healthy lifestyle factors affecting the development of PCa.

**Methods for data acquisition:**

The literature focusing on modifiable risk factors for prostate cancer was reviewed. Medline and Embase via the Ovid database were searched, and all relevant and inherent articles were analyzed. Non-eligible publications, such as review articles, editorials, comments, guidelines, or case reports, were excluded.

**Synthesis of the evidence obtained from data analysis:**

This review confirms that there is strong evidence that being overweight or obese increases the risk of advanced prostate cancer (assessed by body mass index (BMI), waist circumference, and waist–hip ratio), particularly considering central adiposity and ethnicity as contributing factors. The possible contribution of smoking still seems not entirely clear, while alcohol seems to affect PCa prevention in patients taking 5α-reductase inhibitors (5-ARIs). Dietary fibers may have anti-inflammatory properties and improve insulin sensitivity by reducing IGF bioactivity. In particular, dietary fiber intake like insoluble and legume fibers may be inversely associated with prostate cancer risk. Also, hyperglycemia and hyperinsulinemia, with untreated diabetic fasting blood glucose levels, could be modifiable prostate cancer risk factors. In two studies, aspirin was associated with a lower risk of lethal PCa and overall mortality. Concerning the role of vitamins, despite conflicting and divergent results, serum retinol concentration seems to be associated with an increased risk of prostate cancer and high-grade prostate cancer. Some studies resulted in disagreement about the role of selenium and vitamin E. We found inconsistent and controversial findings about the association between vitamin D and prostate cancer risk.

**Conclusion:**

Far from being conclusive evidence, our findings confirm some strong evidence already found in the previous literature and highlight the need to clarify the role of some risk factors whose role is not yet completely known. This effort would facilitate the cultural and social change that may allow the shift from the treatment of prostate cancer when diagnosed to the real efforts needed for systematic prevention.

## 1 Introduction

Prostate cancer (PCa) is the most common cancer among men worldwide (1), with the highest incidence and the third highest mortality rate in Europe (2). The advent of prostate-specific antigen (PSA) and its derivatives increased the number of patients being diagnosed with PCa. Non-modifiable risk factors, such as age, ethnicity, and hereditary factors, represent well-established causes underlying the etiology of PCa (3, 4). Nevertheless, the impacts of modifiable risk factors, such as diet, physical activity, obesity, and smoking, remain largely unclear (5).

In 2018, the World Cancer Research Fund (WCRF) and the American Institute for Cancer Research (AICR) presented the Continuous Update Project expert report on prostate with the aim to analyze cancer prevention through diet, nutrition, and physical activity. The recommendations of this report stated evidence on obesity, highlighting that waist circumference and waist–hip ratio are strongly associated with an increased risk of advanced prostate cancer, defined as high-grade PCa (Gleason score ≥7) or stage 3–4 on the American Joint Committee on Cancer (AJCC 1992) classification. Also, there is strong evidence that beta-carotene is unlikely to have a role in the carcinogenesis of PCa. Despite this robust and strongly supported evidence, only limited and inconsistent evidence is reported in the literature about other modifiable risk factors (6, 7).

Considering the impossibility to manage non-modifiable risk factors, a clear analysis of the modifiable and preventable ones appears of utmost importance. In this review, we aimed to summarize the most recent evidence about the impact of diet, habits, physical activity, and other modifiable risk factors on the carcinogenesis of PCa.

## 2 Methods for data acquisition

### 2.1 Search strategy

The current review was carried out in accordance with the Preferred Reporting Items for Systematic Reviews and Meta-Analyses (PRISMA) statement (8).

Medline and Embase via Ovid database were searched using the following keywords (“prostate cancer” OR “prostatic cancer” OR “prostatic tumor” OR “prostatic adenocarcinoma”) AND (“lifestyle” OR “BMI” OR “alcohol intake” OR “prevention” OR “obesity” OR “physical activity” OR “exercise” OR “healthy” OR “smoking” OR “sedentary” OR “pollution” OR “food” OR “diet” OR “nutrition” OR “antioxidant” OR “sex” OR “sexual activity” OR “infections” OR “testosterone” OR “hormones” OR “androgens” OR “nutrients” OR “metabolic syndrome”).

The literature search was performed in February 2023. Only articles published in the last 10 years were considered. Two authors independently selected the articles for inclusion in the review. When there was no agreement between the authors for inclusion/exclusion, a third author was involved in the definitive decision.

**Figure 1:**
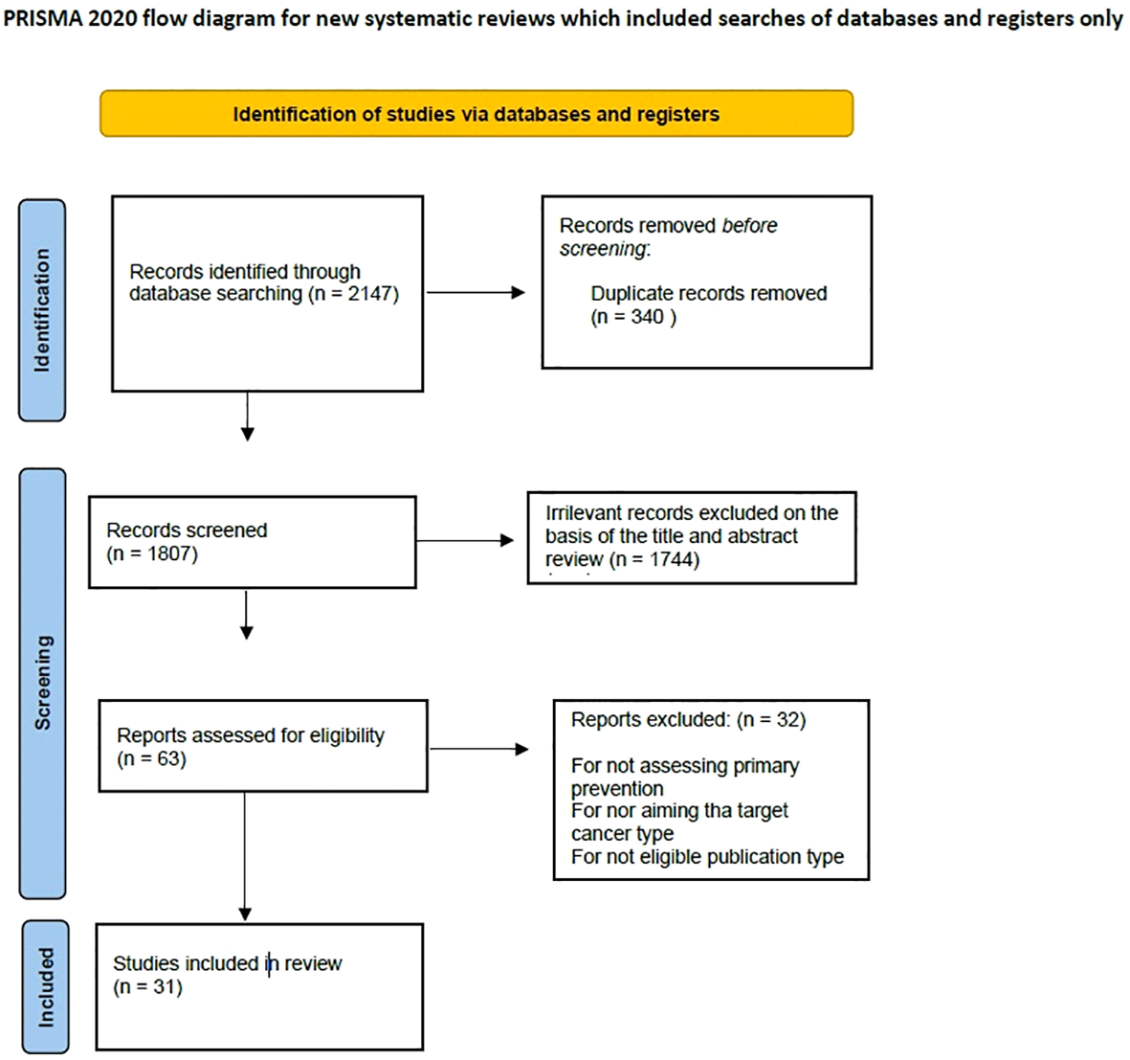
Literature search and selection.

The literature search and the selection process are reported in **Figure 1**.

### 2.2 Study selection

There was no restriction on the type of study (retrospective or prospective), but only clinical trials and randomized controlled trials were considered, excluding non-eligible publication types, such as review articles, comments, guidelines, editorials, and case reports.

A total of 2,147 articles were identified and screened through the evaluation of the title and the abstract to exclude irrelevant studies. When the abstract clearly showed that the related article did not focus on the objective of the present review, it was excluded. In case of doubt, the article was studied for potential inclusion. Subsequently, full-text assessment was performed for all articles included. Articles were excluded when results did not achieve statistical significance and/or when statistical analysis was not robust. Duplicates (defined as articles clearly reporting the same series) and irrelevant articles, which would not contribute to the review, were excluded. Only articles in English were considered.

At the end of the selection process, 31 articles were included in the review (**Table 1**).

**Table 1:** Synthesis of the evidence about the association between modifiable risk factors and prostate cancer.

Lifestyle habits: tobacco and alcohol consumption, sexual activity, and DUD				
Study	Study design, population	Number of patients	Results	Main findings
Zuccolo et al. (2013) (9)	Case–control study nested within ProtecT trial UK	2,386 men aged 50–69 with histologically confirmed PCa and 12,727 controls	Increasing quantity and frequency of alcohol consumption (10 units/week) were associated with lower PSA (Ratio of Geometric Means (RGM) 0.98; 95% confidence interval [CI], 0.98–0.99) and decreased risk of low Gleason grade tumors (Relative Risk Ratio (RRR) 0.96; 95% CI, 0.93–0.99) and with a small increase for the more aggressive high-grade tumors (RRR 1.04; 95% CI, 0.99–1.08).	Small increase in risk of high-grade prostate cancer.
Fowke et al. (2014) (10)	Cohort nested within REDUCE trial Europe, Canada, the UK, Puerto Rico, and others	6,274 REDUCE participants: 988 with low-grade PCa (Gleason < 7) and 435 men with diagnosed high-grade PCa (Gleason 7)	Approximately 49% and 26% of participants were categorized as moderate (14 drinks/week) and heavy drinkers (>14 drinks/week), with increased risk of high-grade PCa (odds ratio [OR], 1.67; 95% CI, 1.15–2.45; *p* < 0.01; and OR, 1.60; 95% CI, 0.98–2.51; *p* = 0.06, respectively). Dutasteride intake was associated with a significant reduction in total and low-grade PCa (OR, 0.67; 95% CI, 0.58–0.77; *p* < 0.001) and with a reduced risk of high-grade PCa only among the abstainers (OR, 0.59; 95% CI, 0.38–0.90; *p* = 0.015).	The protective association between dutasteride and high-grade PCa was reduced by increasing alcohol intake.
Chhim et al. (2015) (11)	Prospective observational study France	2,771 men participated in the Supplementation en Vitamines et Minéraux Antioxydants study	123 prostate cancers were diagnosed. Alcohol intake was directly associated with PCa among individuals with low dietary fiber intake (hazard ratio [HR], 1.37; 95% CI, 0.65–2.89; *p* for trend = 0.02), but not among controls with higher dietary fiber intake (*p* for trend = 0.6). *p*-Interaction between alcohol and dietary intake was statistically significant (*p* = 0.01).	Dietary fiber intake can modulate the association between alcohol intake and risk of prostate cancer.
Ho et al. (2014) (12)	Cohort nested within REDUCE trial Europe, Canada, the UK, Puerto Rico, and others	6,420 REDUCE participants: 2937 never smokers, 2554 ex-smokers, and 929 current smokers	941 men had cancer at the first on-study biopsy. Among these, current and also former smokers were not significantly associated with total or low-grade PCa (all *p* > 0.36). Current smokers were instead at increased risk of high-grade disease (OR, 1.44; *p* = 0.028) compared with former smokers (OR, 1.21; *p* = 0.12).	Smoking was unrelated to overall PCa diagnosis but was associated with increased risk of high-grade PCa.
Dahlman et al. (2022) (13)	Retrospective and prospective study Swedish	1,371,268 men from national register. Of these, 9,259 were registered with DUD	DUD was associated with a slightly increased risk of incident prostate cancer (HR: 1.07; 95% CI, 1.00–1.14; *p* = 0.048) and higher risk of fatal prostate cancer (HR, 1.59; 95% CI, 1.40–1.82; *p* < 0.001).	DUD is reported to increase the risk of fatal prostate cancer possibly related to undertreatment in this population.
Spence et al. (2014) (14)	Case–control study (PROtEuS) Canada	1,590 cases of PCa and 1,628 controls	To have more than 20 sexual partners during lifetime was associated with a decreased risk of overall and low-grade PCa (OR, 0.78; 95% CI, 0.61–1.00; and OR, 0.75; 95% CI, 0.57–0.99, respectively).	Reduction of risk of prostate cancer in men with an elevated number of sexual partners.
Rider et al. (2016) (15)	Cohort nested in HPFS	31,925 men	3,839 men with PCa diagnosis. Elevated ejaculatory frequency (≥21 per month) at the ages of 20–29 and 40–49 years was found to be positively associated with a decreased risk of PCa (HR, 0.81; 95% CI, 0.72–0.92; *p* < 0.0001; and HR, 0.78; 95% CI, 0.69–0.89, respectively).	Positive role of frequent ejaculation during lifetime in PCa prevention.
Cirakoglu et al. (2018) (16)	Observational study Turkey	317 patients who underwent prostate biopsy	171 patients with benign biopsy results and 146 patients with PCa were identified. 66.7% of the benign group had only one sexual partner versus 40.6% of the PCa group. Median number of sexual partners was 1 ± 4 (1–100) in the benign group and 2 ± 6 (1–500) in the malignant group (statistically significant *p* = 0.039).	Association between the number of sexual partners during lifetime and incidence of PCa.
Diet and chronic inflammation				
Brasky et al. (2013) (17)	Case–cohort design nested within SELECT trial Europe, Canada, the UK, Puerto Rico, and others	684 participants with low-grade PCa, 156 with high-grade PCa, and 1,364 controls	Men with high concentrations of LCω-3PUFA had increased risks for low-grade (HR, 1.44; 95% CI, 1.08–1.93), high-grade (HR, 1.71; 95% CI, 1.00–2.94), and total prostate cancer (HR, 1.43; 95% CI, 1.09–1.88).	This study confirms an increased prostate cancer risk among men with high blood concentrations of serum phospholipid long-chain ω-3 fatty acids.
Deschasaux et al. (2014) (18)	Cohort nested within SU.VI.MAX France	Cohort of 3,313 participants, 139 prostate cancer cases during a median follow-up of 12.6 years	Prostate cancer risk was inversely associated with total dietary fiber intake (HR of quartile 4 *vs.* quartile 1, 0.47; 95% CI, 0.27–0.81; *p* = 0.001), insoluble (HR, 0.46; 95% CI, 0.27–0.78; *p* = 0.001), and legume (HR, 0.55; 95% CI, 0.32–0.95; *p* = 0.04) fiber intakes. No association between prostate cancer risk and soluble, vegetable, fruit, and cereal fiber intakes was detected.	Dietary fiber intake (such as total and insoluble or from legumes) was inversely associated with prostate cancer risk.
Bashir et al. (2015) (19)	Case–control study Pakistan	102 cases of prostate cancer and 204 controls	Consumption of red meat was strongly (OR, 3.413; 95% CI, 1.464–7.959; *p* = 0.004) associated with increased risk of prostate cancer. Also, the consumption of fat items was related to a major risk (OR, 2.454; 95% CI, 1.171–5.145; *p* = 0.017). Fluid, vegetable, and fruit intake instead was associated with a decreased risk of prostate cancer (OR and corresponding 95% CI of 0.21; 0.10–0.44, 0.10; 0.05–0.19 and 0.09; 0.03–0.23, respectively).	Diet may play a role in protecting against prostate cancer.
De Stefani et al. (2016) (20)	Case–control study Uruguay	464 cases of prostate cancer and 472 controls	Increased consumption of total meat (OR, 5.19; 95% CI, 3.46–7.81), red meat (OR, 4.64, 95% CI, 3.10-6.95), and processed meat (OR, 1.78; 95% CI, 1.22–2.59) was associated with increased risk of prostate cancer. The effects were stronger among obese patients. All the nutrients increased the risk of prostate cancer with a significant dose–response. The strongest association was observed for cholesterol (OR, 5.61, 95% CI, 3.75–8.50; *p* for trend <0.0001).	Red meat and meat nutrients may play a role in the etiology of prostate cancer.
Diallo et al. (2018) (21)	Prospective study of the French NitriNet-Santé cohort (61476 participants) France	222 prostate cancer cases (88% Gleason score <7)	Red meat intake was associated with increased risk of overall cancers (HR Q5 *vs.* Q1, 1.31 (1.10–1.55); *p* for trend = 0.01), but no association was observed between red meat and processed meat intake and prostate cancer risk (*p* for trend = 0.9).	Consumption of red meat seems not to be involved in the carcinogenesis of prostate cancer.
Miles et al. (2018) (22)	Cohort nested within PLCO cancer Screening Trial (22720 participants) UK	1,996 men with prostate cancer	Increased consumption of added or concentrated sugars in beverages (90 kcal/day) was associated with an increased risk of prostate cancer in the highest quartile of sugar consumption (HR, 1.21; 95% CI, 1.06–1.39; *p* < 0.01). There were no linear associations with the consumption of sugars from desserts and juice.	Positive association between sugar-sweetened beverages and an increased risk of prostate cancer.
Murtola et al. (2018) (23)	Cohort nested within FinRSPC trial Finland	17,860 participants with 1,663 cases of PCa	Men with diabetic high blood glucose levels had increased risk of PCa, for all tumor grades, (HR 1.52; 95% CI, 1.31–1.75), especially compared to normoglycemic men. Also, antidiabetic drugs used can remove the risk association.	High diabetic blood glucose levels and untreated diabetes may constitute a prostate cancer risk factor
BMI and metabolic syndrome				
Nemesure et al. (2012) (24)	Case–control study from PCBP study (2002–2011) Barbados	963 prostate cancer cases and 941 controls	In this case–control study, current weight and BMI were not related to prostate cancer risk (OR, 1.00; 95% CI, 1.00–1.00; and OR, 1.00; 95% CI, 0.98–1.02). Nevertheless, they found a positive association with increment of waist size as measure of central adiposity (OR, 1.84; 95% CI, 1.19–2.85 a greater risk for the fourth quartile). Also, waist–hip ratio was strongly and positively associated with prostate cancer.	Measures of central rather than global adiposity may be more predictive of prostate cancer.
Kayali et al. (2014) (25)	Case–control study from Turkey	170 patients who underwent prostate biopsy	Patients were divided into four groups according to the presence or absence of metabolic syndrome (MetS) and late-onset hypogonadism (LOH). In the group with MetS and LOH together, 37.5% of patients developed PCa, with a significant difference in terms of detection of PCa and aggressive PCa compared with the group with neither MetS nor LOH (37.5% *vs.* 18.2%, *p = 0.031 and 21.9% *vs.* 6.5%, *p = 0.019).	MetS and LOH together can represent a higher risk for PCa, especially for aggressive forms.
Liang et al. (2014) (26)	Cohort nested within SELECT trial Europe, Canada, the UK, Puerto Rico, and others	3,258 patients who underwent biopsy, including 1,902 men with PCa	Increased BMI was not associated with higher risk of PCa overall but was significantly associated with higher risk of HGPCa (i.e., Gleason score ≥7 prostate cancer) (OR, 2.31; *p* = 0.03) in patients with negative familiarity. Men with a positive family history of PCa had rapidly increased risks of PCa and HGPCa as BMI increased (PCa: OR, 3.73; *p* = 0.02; HGPCa: OR, 7.95; *p* = 0.002).	BMI represents an independent predictive risk factor of PCa and HGPCa.
Vidal et al. (2014) (27)	Cohort nested within REDUCE trial Europe, Canada, the UK, Puerto Rico, and others	6,427 patients who underwent at least one biopsy: 1,739 men with normal weight; 3,384 overweight and 1,304 obese	Higher BMI was associated with a lower PSA (*p* = 0.06). 1,448 (23%) cases of prostate cancer were found (7%). In a multivariable analysis, obesity was found to be not related to overall PC risk (OR, 0.92; 95% CI, 0.79–1.07; *p* = 0.28) but was significantly associated with lower risk of low-grade PCa (OR, 0.79; 95% CI, 0.65–0.94; *p* = 0.01) and a higher risk of high-grade PCa (OR, 1.28; 95% CI, 1.01–1.63; *p* = 0.04).	Obesity and a higher BMI may have a role in the carcinogenesis of aggressive forms of PCa.
Boehm et al. (2015) (28)	Case–control study North America	1,933 incident PCa cases	A waist circumference ≥102 cm and a waist–hip ratio >1.0 were associated with an increased risk of high-grade PCa (Gleason score ≥7), when adjusting for BMI. Conversely, an elevated BMI ≥ 30 kg/m^2^ seemed to have a lower risk of PCa, independently from the grade.	This suggests that at higher BMI value, a specific body fat distribution (central adiposity) is more directly related to an increased risk of PCa development (where BMI alone is not).
Choi et al. (2018) (29)	Nationwide study Korea	10,516,985 male subjects form the NHIC between 2009 and 2012	2,002,375 (19%) and 2,629,858 (25%) subjects with non-alcoholic fatty liver disease (NAFLD) were identified based on fatty liver index (FLI) and hepatic steatosis index (HSI), respectively. Both FLI ≥ 60 and HSI ≥ 36 were associated with the development of PCa (HR 1.09; 95% CI, 1.06–1.12; and HR 1.19; 95% CI, 1.16–1.23). In particular, the risk of developing PCa was greater in non-obese persons with NAFLD than those with a BMI ≥ kg/m^2^.	NALFD, defined by FLI and HSI, can be associated with an increased risk of PCa.
Jamnagerwalla et al. (2018) (30)	*Post-hoc* analysis from the REDUCE trial Europe, Canada, the UK, Puerto Rico, and others	4,974 subjects not using statins	High total serum cholesterol was associated with an increased risk of high-grade prostate cancer diagnosis (OR, 1.05; 95% CI, 1.00-1.09; *p* = 0.048); instead, it was not related to either overall PCa or low-grade prostate cancer risk (*p* > 0.185). No association was found instead with serum LDL levels (*p* > 0.137). In contrast, high levels of serum HDL were associated with an increased risk of overall PCa (OR, 1.08; 95% CI, 1.01–1.16; *p* = 0.033) and high-grade PCa (OR, 1.14; 95% CI, 1.01–1.28; *p* = 0.034).	High total serum cholesterol and high HDL serum level were associated with an increased risk of high-grade PCa
Use of hypertensive drugs, NSAIDs, and aspirin				
Veitonmaki et al. (2013) (31)	Case–control study Finland	24,657 case–control pairs	Non-steroidal anti-inflammatory drug (NSAID) users had greater overall and high-grade prostate cancer risk than the non-users (53.6% *vs.* 46.4%, respectively; OR, 1.3; 95% CI, 1.3–1.4; and 14.1% *vs.* 11.8%; OR, 1.6; 95% CI, 1.5–1.8). The risk increased similarly for each type of NSAID used, with the exception of aspirin (8.1% of the cases and 7.9% of the controls), which represented a protective factor for the overall PCa risk (OR, 0.90; 95% CI, 0.84, 0.96).	Aspirin use seems to be associated with a decreased overall prostate cancer risk.
Downer et al. (2017) (32)	Cohort nested within PHS trial USA	22,071 patients	502 men with developed lethal prostate cancer. Current (HR, 0.68; 95% CI, 0.52–0.89) and past (HR, 0.54; 95% CI, 0.40–0.74) regular aspirin intake (>3 tablets for a week) were associated with a lower risk of lethal PCa, compared to never users. Also, current post-diagnostic aspirin intake was associated with a lower risk of lethal prostate cancer (HR 0.68; 95% CI, 0.52–0.90) and overall mortality (HR 0.72; 95% CI, 0.61–0.9).	A regular aspirin intake was associated with a lower risk of lethal PCa and improved survival.
Siltari et al. (2020) (33)	Prospective study Finland	(8,253 patients)	On the whole, anti-hypertensive drugs correlated with death from prostate cancer before diagnosis (HR, 1.21; 95% CI, 1.04–1.4) and after diagnosis (HR, 1.2; 95% CI, 1.02–1.41). Angiotensin II type 1 receptor blockers were associated with improved survival (HR, 0.81; 95% CI, 0.67–0.99), while diuretics were associated with an increased risk of mortality (HR, 1.25; 95% CI, 1.05–1.49).	ATr blockers correlated with improved survival, while diuretics correlated with an increased risk of mortality from prostate cancer.
Thyroxine, thyroid-stimulating hormone, and hypothyroid status				
Mondul et al. (2012) (34)	Prospective study within the ATBC Study Finland	402 male smokers	High serum TSH level was associated with a decreased risk of prostate cancer compared to men with lower TSH (Q5 *vs.* Q1–4: OR, 0.70; 95% CI, 0.51–0.97; *p* = 0.03). Comparing T4 and TSH identifies men as hypothyroid, euthyroid, hyperthyroid, or hypothyroid, which resulted in a lower risk of PCa compared to euthyroid men (OR, 0.48; 95% CI, 0.28–0.81; *p* = 0.006).	Elevated serum TSH and hypothyroid condition represent were associated with a decreased risk of prostate cancer.
Vitamins and micronutrients				
Albanes et al. (2014) (35)	Case–cohort sample derived from SELECT trial Europe, Canada, the UK, Puerto Rico, and others	1,746 prostate cancer cases and 3,211 controls from SELECT trial	High α-tocopherol concentrations were associated with a similar risk to lower concentrations [overall HR for fifth (Q5) *vs.* first quintile (Q1), 1.21 (95% CI, 0.88–1.66; *p* for trend = 0.24; in the trial placebo arm, Q5 HR, 0.85; 95% CI, 0.44–1.62; *p* for trend = 0.66]. Nevertheless, there was a strong positive plasma α-tocopherol association among men receiving the trial selenomethionine supplement [Q5 HR, 2.04; 95% CI, 1.29–3.22; *p* for trend = 0.005], limited to high-grade disease (Gleason grade 7–10, overall Q5 HR, 1.59; 95% CI, 1.13–2.24; *p* for trend = 0.001; among men receiving selenomethionine, HR, 2.12; 95% CI, 1.32–3.40; *p* for trend = 0.0002).	Evidence of an interaction between α-tocopherol and selenium itself or selenomethionine increasing high-grade prostate cancer risk.
Schenk et al. (2014) (36)	Cohort nested within PCTP	1,695 prostate cancer cases and 1,682 controls	No evidence of associations between serum 25(OH)D and overall prostate cancer risk and an inconsistent correlation for Gleason 2–6 cancers. For Gleason 8–10 prostate cancers, 25(OH)D concentrations were associated with a linear decrease in risk instead (quartile 4 *vs.* 1: OR, 0.55; 95% CI, 0.32–0.94; *p* for trend = 0.04).	Higher serum 25(OH)D may substantially reduce high-grade prostate cancer risk.
Nash et al. (2015) (37)	Case–control study nested in the PCPT trial	975 cases/1,009 frequency-matched controls in the placebo arm and 708 cases/743 frequency-matched controls in the finasteride arm	Serum retinol concentrations were associated with an increased risk of overall prostate cancer [OR (95% CI) comparing the highest quartile of serum retinol to the lowest: 1.30 (1.00, 1.68)] and high-grade prostate cancer [OR (95% CI): 1.74 (1.14, 2.68)] in the placebo arm of the trial only. Also, in the placebo arm, there was a moderate positive association of α-carotene with the risk of total prostate cancer [OR (95% CI): 1.32 (1.01, 1.73)]. No associations were observed for retinol and carotenoids in the finasteride arm.	In the placebo arm, high serum retinol and α-carotene concentrations were associated with an increased risk of total and high-grade PCa.
Antwi et al. (2016) (38)	Population-based case–control study North America	1,023 cases of African-Americans (AA) and 1,079 cases of European-Americans (EA)	Higher intake of lycopene among EAs and higher β-cryptoxanthin intake among AAs. Dietary lycopene was associated inversely with high aggressive PCa among EAs (OR, 0.55; 95% CI, 0.34–0.89; *p* for trend = 0.02), while an inverse association was observed between dietary β-cryptoxanthin intake and high aggressive PCa among AAs (OR, 0.56; 95% CI, 0.36–0.87; *p* for trend = 0.01).	High lycopene and β-cryptoxanthin intake may represent a protective risk factor against aggressive PCa.
Morgia et al. (2017) (39)	*Post-hoc* analysis of the Procomb trial (Italy)	Group A: 134 patients treated with Se and Ly Group B: 75 controls	During a follow-up time of 2 years, 9 patients (4.3%) were diagnosed with PCa, and 15 (7.2%) were diagnosed with BPH. There were no statistical differences in terms of mean changes in PSA and Gleason scores between the two groups. Among the patients treated with Se and Ly, there was an RR of 1.07 for PCa and an RR of 0.89 among group B (*p* = 0.95). Supplementation with Se and Ly was not associated with greater risk of PCa (HR, 1.38; *p* = 0.67).	No evidence of detrimental role of selenium and lycopene supplementation in increasing PCa risk.
Hada et al. (2020) (40)	Cohort nested within ATBC study	29,104 patients	Higher serum retinol was not associated with overall cancer risk (highest *vs.* lowest quintile: HR, 0.97; 95% CI, 0.91–1.03; *p* for trend = 0.43). Nevertheless, higher retinol concentrations were associated with increased risk of prostate cancer (highest *vs.* lowest quintile: HR, 1.28; 95% CI, 1.13–1.45; *p* for trend <0.0001).	High serum retinol was associated with an increased risk of prostate cancer.
Hoyt et al. (2019) (41)	Cohort nested within PLCO screening trial USA	28,356 men who completed DQX and 48,090 men who completed DHQ	Dietary intakes of phylloquinone, menaquinones, and total vitamin K, assessed with either the DQX or DHQ, were not significantly associated with the risk of advanced, non-advanced, and total prostate cancer.	No evidence that vitamin K intake influences the occurrence of total and high-grade prostate cancer.

### 2.3 Data extraction

Two authors extracted the following data from the included articles: year of publication, type of the study, funding, patients’ characteristics, and results.

As quantitative analysis was not possible due to the very high heterogeneity among the included studies, only qualitative analysis was carried out.

### 2.4 Studies included

All studies included were published between 2012 and 2023. A total of 21 studies were nested case–control (NCC), three studies were observational, and seven were case–control studies.

The cohort was nested within the following trials:

◼ The Selenium and Vitamin E Cancer Prevention Trial (SELECT): a randomized placebo-controlled trial that tested the role of selenium and vitamin E in reducing prostate cancer risk (the USA, Puerto Rico, and Canada) (42).
◼ The Prostate Testing for cancer and Treatment (ProtecT) study: a multicenter randomized controlled trial (UK) (43).
◼ The Alpha-Tocopherol, Beta-Carotene Cancer Prevention (ATBC) Study: a randomized, double-blind, placebo-controlled trial on the effects of supplementation with α-tocopherol and β-carotene on cancer incidence (USA and Finland) (44).
◼ The Reduction by Dutasteride of Prostate Cancer Events (REDUCE) study: a multicenter, randomized, double-blind, placebo-controlled study comparing dutasteride with placebo (Europe, Canada, UK, Puerto Rico, and others) (45).
◼ The Prostate Cancer Prevention Trial (PCPT): a randomized, placebo-controlled trial testing the role of 5α-reductase inhibitor in the prevention of prostate cancer (USA) (46).
◼ The Supplementation en Vitamines et Minéraux Antioxydants (SU.VI.MAX) cohort: a randomized, double-blind, placebo-controlled trial assessing the effect of daily supplementation of antioxidants on the incidence of cardiovascular diseases and cancers (France) (47).
◼ The Physicians’ Health Study (PHS I): a randomized, placebo-controlled trial on aspirin and β-carotene for the prevention of cardiovascular disease and cancer (USA) (48).
◼ The Prostate, Lung, Colorectal and Ovarian (PLCO) Cancer Screening Trial: a large, prospective, randomized, multi-site study (USA) investigating the effects of cancer screening on cancer mortality (49).
◼ The Finnish Randomized Study of Screening for Prostate Cancer (FinRSPC), the largest component of the multinational European Randomized Study of Screening for Prostate Cancer (ERSPC), whose main goal was to find out if systematic screening can decrease prostate cancer mortality (50).
◼ The NutriNet-Santé study was an ongoing web-based cohort launched in France in 2009 with the aim to study the association between nutrition and health (51).

In terms of geographical distribution, many studies were multicentric randomized studies including patients from the USA, the UK, Europe, Canada, and Puerto Rico, with a small contribution from Finland, France, Pakistan, Uruguay, Turkey, Korea, and Italy.

The results of the studies included are summarized in **Table 1**.

## 3 Synthesis of the evidence obtained from data analysis

### 3.1 Lifestyle habits

#### 3.1.1 Tobacco consumption

Cigarette smoking is considered a major public health concern worldwide due to its consequences in terms of mortality and morbidity. Despite the correlation between smoking and several solid tumors, the relationship between smoking and PCa remains a matter of debate (52). The REDUCE trial offered an opportunity to evaluate the relationship between cigarette smoking and prostate cancer. Ho et al. (12) conducted a logistic regression to test the association between smoking and cancer on the first on-study biopsy in REDUCE. Among men with high PSA levels and negative biopsy in REDUCE, smoking was unrelated to overall prostate or low-grade PCa. However, smoking was found to be associated with an increased risk of high-grade PCa.

#### 3.1.2 Alcohol consumption

Concerning alcohol consumption, most of the evidence in the literature confirms a correlation with the increased risk of high-grade prostate cancer, with a smaller influence on the overall risk. In particular, alcohol consumption among moderate and heavy drinkers (estimated at least 10 units/week) appears to increase the risk of high-grade PCa (9). Fowke et al. (10) confirmed that alcohol affects PCa in patients on 5α-reductase inhibitors (5-ARIs). Dutasteride was assumed to be associated with a significantly reduced risk of high-grade PCa among alcohol abstainers, suggesting that alcohol intake represents an unfavorable risk factor in those taking phosphodiesterase inhibitors. However, it is reported that the intake of a high amount of dietary fiber represents a protective factor among alcohol consumers (11).

#### 3.1.3 Sexual habits/activity

The role of sexual activity as a modifiable risk factor in PCa prevention is still being debated. The main studies in the literature focused on ejaculatory frequency, number of sexual partners, and age of the first intercourse. Sexually transmitted infections (STIs) have also been investigated. However, it was not possible to unequivocally clarify the correlation with the carcinogenesis of prostate cancer (53). Spence et al. (14) carried out a case–control study, finding a reduction in the risk of overall and low-grade PCa among men with multiple female sexual partners during their lifetime (more than 20). In contrast, men with multiple male sexual partners had a slightly increased risk, with an apparent association with a higher risk of STIs. Frequent sexual intercourse may lead to a potentially increased exposure to sexually transmitted diseases (STDs) and is associated with higher androgen function and higher PCa risk (54). Indeed, Cirakoglu et al. (16) found a positive association between the number of sexual partners during a lifetime and the incidence of PCa. In addition, Rider et al. (15) supported the “prostate stagnation hypothesis”, as they reported a positive role of frequent ejaculation during a lifetime (more equal than 20 per month) in preventing prostate cancer. This can be related to the decrease of carcinogenic molecules within prostatic fluid due to frequent ejaculation.

### 3.2 Diet and chronic inflammation

Dietary fiber intake is associated with anti-inflammatory properties through a decreased oxidation of lipids and better control of glucose serum levels, which are known for decreasing proinflammatory cytokines like plasma interleukins (55). Chronic inflammation contributes to several forms of cancers, and it is well known that this can stimulate prostate carcinogenesis (56, 57). Deschasaux et al. confirmed that high dietary fiber is inversely associated with PCa risk, especially for insoluble and legume fiber intake. However, the authors found no association between prostate cancer risk and soluble, cereal, vegetable, and fruit fiber intakes.

Phospholipid long-chain ω-3 fatty acids represent serum biomarkers of fatty acid intake, whose role in prostate cancer carcinogenesis has been recently suggested (58).

Brasky et al. (17) investigated the association between plasma LCω-3PUFA and PCa, confirming an increased prostate cancer risk.

#### 3.2.1 Meat

The International Agency for Research on Cancer (WHO-IARC) classified processed meat intake as “carcinogenic to humans” and consumption of red meat as “probably carcinogenic to humans” (59). The carcinogenic role of processed and red meat was confirmed also in two case–control studies (19, 20), in which increased consumption was directly associated with increased risk of PCa, with a significant dose–response rate. Moreover, Bashir et al. (19) found a decreased risk association with fluid, vegetable, and fruit intake. Nevertheless, Diallo et al. (21) observed no correlation between red or processed meat intake and PCa but found that meat intake is involved in carcinogen-induced tumors such as colorectal cancer.

#### 3.2.2 Diabetes

Glucose metabolism and chronic inflammation may have a role in PCa development (60), modulating signaling pathways and promoting uncontrolled cell proliferation and oxidative stress (61). Murtola et al. (23) evaluated prostate cancer risk in relation to normal and elevated serum glucose levels in diabetic patients. Diabetic patients with high blood glucose levels have an increased risk of overall PCa. The use of antidiabetic drugs and glucose level normalization is reported to contribute to decreasing the risk of PCa.

Miles et al. (22) studied its relationship with concentrated dietary sugar intake. Increased consumption of added or concentrated sugar-sweetened beverages (in the highest quartile) is related to an increased risk of PCa. There is a non-linear association instead with sugar intake from juices and desserts, suggesting a role of low glycemic index food selection in the prevention.

#### 3.2.3 Body mass index and metabolic syndrome

The relationship between obesity, body mass index (BMI), and PCa has been extensively investigated in literature and was confirmed by the WCRF/AICR recommendations in 2018. In particular, obesity is related to lower concentrations of free testosterone and a decreased risk of localized/low-grade PCa and with an increased risk of advanced/high-grade PCa (62, 63). In two nested case–control studies from the SELECT and REDUCE trials (26, 27), increased BMI was found to be associated with a higher risk of high-grade PCa (Gleason score ≥7) in patients uninformed about PCa.

Nemesure et al. (24), in a case–control study on the Barbados population, found that a major correlation between waist size and waist–hip ratio is a measure of central adiposity and adiposity distribution, and prostate cancer risk. These results were confirmed also in another case–control study carried out by Boehm et al. (28). This suggests that body fat distribution may be more directly related to an increased high-grade risk PCa than BMI alone.

Abdominal obesity is closely related to metabolic syndrome (MetS), including insulin resistance, hypertension, impaired glucose tolerance, and lipid profile (64). According to some authors, late-onset hypogonadism (LOH), defined as the decrease of androgen levels, should be considered part of the MetS (65, 66). Kayali et al. (25) published a case–control study on the Turkish population subdividing patients into groups according to the presence of MetS and LOH. The authors found that the group with both MetS and LOH had an increased risk of prostate cancer, especially with aggressive histologic features.

In addition, the altered hepatic metabolism in non-alcoholic fatty liver disease (NAFLD), which is associated with metabolic syndrome, determines a suppression of the hepatic glucose leading to hyperglycemia, hypertriglyceridemia, and hyperinsulinemia (27, 67). Choi et al. (29) analyzed the correlation with PCa, suggesting that NAFLD is directly associated with an increased risk of prostate cancer, especially in non-obese patients with NAFLD.

Lastly, Jamnagerwalla et al. (30) found that high total serum cholesterol and high serum HDL are associated with an increased risk of high-grade PCa, thus confirming the importance of metabolic syndrome in the predisposition to PCa.

### 3.3 Use of drugs and vitamins

#### 3.3.1 Use of hypertensive drugs

The use of hypertensive drugs is reported to increase the risk of mortality among patients diagnosed with prostate cancer. In particular, in a Finnish cohort of patients, Siltari et al. (33) found that ACE inhibitors and ATr blockers correlate with improved survival, while diuretics correlate with decreased survival.

Similarly, in a UK longitudinal cohort, Cardwell et al. reported that ACE inhibitors and ATr blockers decreased the risk of cancer-specific mortality (68).

The mechanism related to the role of ACE inhibitors and ATr blockers is unclear but probably involves the activity of angiotensin-II in mediating cell proliferation and fibrosis (69)

#### 3.3.2 Use of aspirin and NSAIDs

Whereas regular aspirin administration probably protects from some malignancies, its exact role in the carcinogenesis of overall PCa and lethal PCa is still unclear. Downer et al. (32) found out that current and previous regular aspirin use (more than 200 mg per week) is associated with a lower lethal prostate cancer risk, especially compared to never users. In this study, the administration of aspirin after diagnosis had an impact on overall mortality. In a Finnish case–control study, Veitonmäki et al. (31) tested the association between non-steroidal anti-inflammatory drugs (NSAIDs) and PCa and found that NSAID users had an increased high-grade and all-grade PCa. This was similar for all types of NSAIDs tested, except for aspirin, which acted as a protective factor for overall prostate cancer risk.

#### 3.3.3 Thyroxine, thyroid-stimulating hormone, and hypothyroid status

Triiodothyronine (T3) and thyroxine (T4) are thought to promote carcinogenesis through cell differentiation and development (54, 70). Mondul et al. (34) explored the relationship between circulating thyroid hormones, thyroid status, and prostate cancer risk. Compared with euthyroid men, the patients with high thyroid-stimulating hormone (TSH) and hypothyroidism had a decreased risk of PCa.

#### 3.3.4 Vitamin and micronutrients

Vitamins are essential nutrients for human metabolism, and they play an important role as coenzymes in the normal functioning of the body as well as in many vital processes. In recent years, it has become clear that vitamins are essential for health and human disease (21, 70), thanks to several studies examining this correlation. However, according to Cancer Research UK, there is no reliable evidence that dietary supplements can help prevent cancer. Evidence in the literature is inconclusive and conflicting, with positive or no reported associations.

The 2018 WCRF/AICR recommendation downgraded lycopene and selenium supplementation from “may reduce prostate cancer risk” to “limited—inconclusive” (7). In fact, Morgia et al. (39) studied the incidence of PCa in a group of patients treated with selenium and lycopene in the previous Procomb study, but the evidence did not support a significant role for supplements in affecting prostate cancer risk. Conversely, Antwi et al. (38) carried out a case–control study among African American (AA) and European-American (EA) men from North America. They observed a higher intake of lycopene among EAs and a higher intake of beta-cryptoxanthin among AAs. Dietary lycopene and beta-cryptoxanthin have an indirect relationship with the development of high-grade PCa among EAs and AAs.

Vitamin E reduces DNA damage, enhances DNA repair, affects cellular responses to oxidative stress, inhibits cell proliferation, enhances immune responses, and reduces cellular testosterone levels (71). Albanes et al. (35) investigated the role of pre-supplementation of plasma α-tocopherol or γ-tocopherol in the development of high-grade (Gleason score 7–10) prostate cancer. Men with higher concentrations of α-tocopherol had a similar risk if compared to men with lower concentrations. Nevertheless, the authors found a strong positive association between plasma α-tocopherol and the risk of developing PCa in men receiving selenomethionine supplementation.

Retinol has been reported to promote cell differentiation and apoptosis. It increases the level of other antioxidants and regulates DNA transcription (63). Nash et al. (37) presented a nested case–control study evaluating the association of serum retinol and carotenoids with overall PCa risk. The authors found that serum retinol levels were associated with an increased risk of all grades of prostate cancer. Even in the placebo group, α-carotene was moderately positively associated with the risk of all grades of prostate cancer. In addition, Hada et al. (40), in 2015, prospectively investigated serum retinol and the overall risk of PCa as well as the role of α-tocopherol and β-carotene in overall PCa prevention. After multivariable adjustment, they concluded that high serum retinol was not associated with overall cancer risk but was associated with an increased risk of prostate cancer (72–74).

Vitamin D metabolites regulate cell growth and differentiation. The administration of vitamin D analogs suppresses prostate cancer progression in animal models and phase II studies (36, 37, 74). Schenk et al. (36) conducted a case–cohort study using data from the PCPT and found that high levels of serum 25(OH)-vitamin D slightly increased the risk of high-grade PCa and could reduce the risk of low-grade PCa.

Hoyt et al. (41) evaluated the relationship between dietary intake of phylloquinone, menaquinone, total vitamin K, and prostate cancer risk. The authors reported clear evidence of the role of vitamin K in the development of prostate cancer. In this study, the authors concluded that vitamin K intake affects the incidence of overall and advanced prostate cancer.

### 3.4 Drug use disorder

Drug use disorder (DUD) is reported to increase the risk of fatal prostate cancer. According to Dahlman et al. (13), DUD gives rise to a little increase in incident prostate cancer, but more interestingly, it is a statistically significant predictor of fatal prostate cancer. The authors hypothesize that in this group of patients, the underlying cause is the more advanced stage at diagnosis due to a delay in the diagnosis and treatment. This study is retrospective and based on the Swedish population.

In a previous study, Chhatre et al. reported that substance use disorders, including prescription drugs and recreational or non-legal substances, are related to an increase in mortality in patients diagnosed with advanced prostate cancer, particularly in patients with mental and behavioral disorders (75).

Similar results are reported by Jayadevappa et al. in a larger longitudinal cohort (76).

## 4 Discussion

In accordance with the latest World Cancer Research Fund International Continuous Update Project report on prostate 2018, a systematic and global analysis of the scientific research on prostate cancer, the present review shed more light on modifiable risk factors of prostate cancer, including diet, micronutrients, obesity, and metabolic syndrome, but may topics remain a matter of debate and controversial (54).

In the last years, many articles have been published on the refinements in the diagnosis of PCa (77) as well as on the algorithms for predicting the risk of recurrence after cutting-edge techniques for the treatment of this disease (78), but no comparable effort has been made in order to implement the strategies to prevent PCa.

Although not conclusive, our review contributes to clarifying potentially modifiable risk factors for the development of PCa and focuses on the role of some biochemical mechanisms not yet fully understood in the pathogenesis of PCa.

One of the major limitations of this review is its failure to quantify the risk for each risk factor considered. Unfortunately, different studies on a specific risk factor use different statistical analyses, so the results are often not comparable. If we found many studies obtaining appreciable results, it was not possible to achieve a cumulative result. As a consequence, for many risk factors, the results of our review could not be conclusive.

In addition, as many authors have highlighted, risk factors are often correlated with each other, thus making it much more difficult, or even impossible, to quantify the role of a single risk factor in the prevention of PCa. In this respect, we argue that policies for the prevention of PCa should not be addressed to a single risk factor, even if important in public health, but to a number of risk factors together, especially when working on lifestyle, diet, and sexual habits.

Noteworthy, major risk factors, such as tobacco, alcohol consumption, and DUD, are risk factors for many other diseases, like lung, liver, and bladder cancers, with well-established evidence in the literature showing that reducing these modifiable risk factors dramatically results in a decrease of the prevalence of these tumors.

Other risk factors concerning lifestyle, diet, and sexual habits are not strictly related to a specific pathology, especially when the reduction of these risk factors is not planned in the scenario of wider prevention policies aiming to improve the general health status. A policy aiming to improve the general health status of patients, through a reduction of modifiable risk factors concerning lifestyle, diet, and sexual habits, would lead to an improvement in quality of life and general health and in turn a decrease in the risk of developing cancer and other non-tumoral pathologies. In this view, PCa would be one of the targets of this policy.

Further studies are needed to reach proper evidence in the perspective that prevention may be effective in limiting the number of patients not undergoing treatment. In this view, the prevention of PCa may improve the quality of life due to the burden of complications and side effects of active treatment, such as surgery, radiotherapy, chemotherapy, and androgen deprivation, which are known for their negative impact on the quality of life.

Last but not least, preventing men from developing prostate cancer would be much more cost-effective for the healthcare systems of the majority of countries. All the efforts to facilitate the shift from the paradigm of treatment of PCa to the paradigm of its prevention (which has been called the “paradigm shift”) would be beneficial, and a deeper insight into the modifiable risk factors of this disease is of the utmost importance to plan policies for systematic prevention (77).

There is common agreement in the literature about the effectiveness of early diagnosis as a policy for cancer prevention. Although a tumor-specific marker for prostate cancer is not available as yet, the advent of PSA and its derivatives, like PSA-IgM, as well as other markers, like PCA-3, has been investigated with the aim to support the early diagnosis of prostate cancer (79–81). Multiparametric MRI has also revolutionized the detection of prostate cancer in its earliest stage (82–85). Unfortunately, overtreatment remains a matter of fact, with many men needing therapy for side effects related to treatment. For this reason, the policies aiming to prevent PCa should focus not only on detecting prostate cancer as early as possible but also on more efforts to the prevention of modifiable risk factors in order to achieve a decrease in the prevalence of prostate cancer and a consequent decrease of the side effects of treatment. This is particularly important when considering the overall costs of PCa.

It goes without saying that this concept of prevention is strictly related to education and cultural environment, which may be different in different countries. In this view, the policies to prevent modifiable risk factors for PCa may be different from country to country and should be made known early to patients.

The present review shows a number of modifiable risk factors that should be known and understood for the implementation of health policies, as well as for counseling patients as regards prostate cancer, particularly those who are at risk due to non-modifiable risk factors, such as familiarity, race, or genetics.

## 5 Conclusion

Prostate cancer is a major issue in men’s health, with an increased number of patients being diagnosed and treated due to the refinements in detection techniques in the last two decades. This led to overtreatment in some cases, unnecessary costs for the health systems, and the need to treat the side effects related to the treatment.

The implementation of policies of prevention may lead to less incidence of PCa. Due to the impossibility to act on non-modifiable risk factors, there is still the chance to apply policies able to decrease the impact of modifiable risk factors.

The present review of the literature showed that many risk factors are fit as targets of prevention policies. Unfortunately, we were not able to quantify the role of every single risk factor due to the fragmentation of the literature or even the presence of conflicting results in some cases. However, focusing on a wide range of risk factors allowed a deeper insight into the mechanisms of nutrients, lifestyle, habits, and drugs in preventing or facilitating PCa. This may support proper patient counseling as well as inform policies for prostate cancer prevention.

## Author contributions

Study concept and design: FZ, UM. Data search and collection: AP, GI. Writing: AP, GG. Editing: AP, DC. Revision: AP, DC, FZ. All authors contributed to the article and approved the submitted version.
